# Oral Piracetam vs Betahistine in Outpatient Management of Peripheral Vertigo; a Randomized Clinical Trial

**Published:** 2019-01-23

**Authors:** Ali Arhami Dolatabadi, Seyedeh Roghieh Larimi, Arash Safaie

**Affiliations:** 1 Department of Emergency Medicine, Imam Hossein Hospital, Medical School, Shahid Beheshti University of Medical Sciences, Tehran, Iran.; 2 Department of Emergency Medicine, Sina Hospital, Tehran University of Medical Sciences, Tehran, Iran.

**Keywords:** Vertigo, piracetam, betahistine, emergency medicine, treatment outcome

## Abstract

**Introduction:**

Although vertigo is a common complaint in patients presenting to the emergency department (ED), its ideal treatment is still under debate. This study was conducted to compare oral betahistine and oral piracetam in management of outpatients with peripheral vertigo.

**Methods:**

This was a randomized clinical trial performed on patients who were presented to the EDs of 4 teaching hospitals, with complaint of true vertigo. Patients were randomly allocated to either betahistine or piracetam group and their 7-day outcomes were compared.

**Results:**

100 cases with the mean age of 54.72 ± 14.09 years were randomly allocated to either group (62.0% female). The two groups were similar regarding age, sex, and intensity of symptom at the time of presenting to the ED. Twelve (24%) patients in piracetam group and 6 (12%) patients in betahistine group experienced adverse events (odds ratio: 2.32, CI 95%: 0.79-6.76; p = 0.125). There were 3 (6%) patients in each group that experienced a recurrence of their symptoms and 2 (4%) patients in each group saw another physician for vertigo.

**Conclusion:**

Oral piracetam is a potentially proper treatment for management of peripheral vertigo and there are few adverse effects associated with it.

## Introduction:

Vertigo is an illusion of movement when there is no motion (1, 2). It is a common complaint in patients presenting to the emergency department (ED) and categorized as peripheral or central vertigo (1-3). Although ideally the underlying cause of vertigo should be treated, peripheral vertigo is usually treated symptomatically. Different drugs from various pharmacologic categories are used to treat peripheral vertigo, including antiemetics, anticholinergics, antihistamines, and calcium channel blockers. Despite the availability of various options for symptomatic treatment of vertigo, a drug of choice has not been defined yet. There are sometimes paradoxical data on the effectiveness of each drug for symptomatic treatment of vertigo (1, 4).

Betahistine is one of the common medications, which is currently used to treat peripheral vertigo. Piracetam is a cyclic derivative of neurotransmitter gamma-aminobutyric acid, which might be effective in treatment of vertigo (5-10). 

By using the combination of betahistine and piracetam in management of peripheral vestibular vertigo Oleg and colleagues concluded that this combination may be more effective than piracetam alone in this regard (11). Intravenous piracetam was as effective as intravenous dimenhydrinate in relieving symptom of vertigo patients in Dugan et al. study (12).

However, the evidence regarding effectiveness of oral piracetam after acute manifestation of peripheral vertigo is sparse. Therefore, this study aimed to compare oral betahistine and oral piracetam in outpatient management of peripheral vertigo.

## Methods:

***Study design and setting***

This randomized clinical trial was conducted on patients who presented to the emergency departments of Imam Hossein, Shohadaye Tajrish, Loghman Hakim and Sina Hospitals, Tehran, Iran, throughout 2016, complaining from true vertigo. All of these hospitals are teaching hospitals.

Ethical approval was obtained from Shahid Beheshti medical University ethics committee (IR.SBMU.SM.REC.1394.25) and the study was registered on Iranian registry of clinical trials with code number: IRCT2016011921063N3. Informed consent was obtained from each patient prior to his or her enrollment in the study.

***Participants***

Patients with acute vertigo who were discharged from the emergency department were included. Exclusion criteria were age below 18 years, prior history of sensitivity to betahistine or piracetam, consumption of drugs which can possibly improve vertigo (antihistamines, benzodiazepines or anticholinergics), history of peptic ulcer disease or pheochromocytoma, patients who did not give consent to participate in the study, patients with central vertigo and patients who were lost to follow-up.

***Intervention***

Patients were enrolled using convenience sampling method and were randomly assigned to either piracetam or betahistine group. Upon discharge, piracetam group patients received 800 mg piracetam tablets (Darou Pakhsh Pharma Chem Co., Tehran, Iran) every 8 hours for 7 days and betahistine group patients received 8 mg betahistine tablets (Betaserc®, Abbott Healthcare SAS, Châtillon-sur-Chalaronne, France) every 8 hours for 7 days. 

Simple randomization was done using a random numbers table, which was generated using Stattrek website “http://stattrek.com/statistics/random-number-generator.aspx”. 

***Outcomes***

The primary outcome was vertigo intensity control 7 days after discharge from ED. The secondary outcomes were nausea and fatigue intensities control 7 days after discharge from ED, drug compliance, and adverse events.

***Data gathering***

Data were acquired using face to face interview with patients at the times of presenting to ED and discharge from ED and using telephone interview 7 days after discharge. Patients’ vertigo, nausea, and fatigue intensities were investigated using 10-point numeric rating scale (NRS). We asked patients about their compliance (taking piracetam or betahistine) using 5-point Likert scale and the occurrence of any complication while taking medications. Patients were asked about the recurrence of their vertigo symptoms, visiting another physician for vertigo or requiring hospitalization because of vertigo during the 7-day period after discharge from ED. An emergency physician and a 3^rd^ year emergency medicine resident were responsible for patients’ allocation and data gathering. 

The prescribing physicians and the patients were not blinded. However, both the physician who interviewed the patients 7 days after their discharge and the statistical analyst were blinded to the prescribed drug.

***Statistical analysis***

We analyzed data using SPSS software version 21 and intention to treat analysis method. Categorical data were analyzed using chi-square or Fisher’s exact tests. Quantitative data were analyzed using t-test, Mann-Whitney or Kruskal-Wallis tests. The sample size of 48 patients in each group was calculated. Failure to control symptoms was defined as score ≥ 3 7 days after taking medicines. P < 0.05 was considered statistically significant.

## Results:

***Baseline characteristics***

One hundred twenty-two patients were assessed for eligibility, out of which 18 cases were excluded for various reasons (flowchart 1). There were 4 (3.8%) cases of loss to follow up. Finally, 100 cases with the mean age of 54.72 ± 14.09 (18-90) years were randomly allocated to each group (62.0% female). Baseline characteristics of the studied patients are compared in table 1. The two groups were similar regarding age, sex, and intensity of symptom at the time of presenting to the emergency department and discharge.

***Outcome***

There were no statistically significant differences between two groups regarding intensity of vertigo, nausea and fatigue 7 days after discharge from ED. Twelve (24%) patients in piracetam group and 6 (12%) patients in betahistine group experienced adverse events (odds ratio: 2.32, CI 95%: 0.79-6.76; p = 0.125). There were 3 (6%) patients in each group who experienced a recurrence of their symptoms and 2 (4%) patients in each group saw another physician for vertigo. The symptoms of one patient in the piracetam group were so severe that required hospitalization on the 7^th^ day after discharge from ED. There was not any significant difference between groups regarding failure to control symptoms.

## Discussion:

Based on the findings, there was not any significant difference between oral betahistine and oral piracetam regarding recurrence, relieving vertigo, nausea, and fatigue symptoms 7 days after discharge from ED. Adverse events in the two groups were comparable as well.

**Figure 1:**
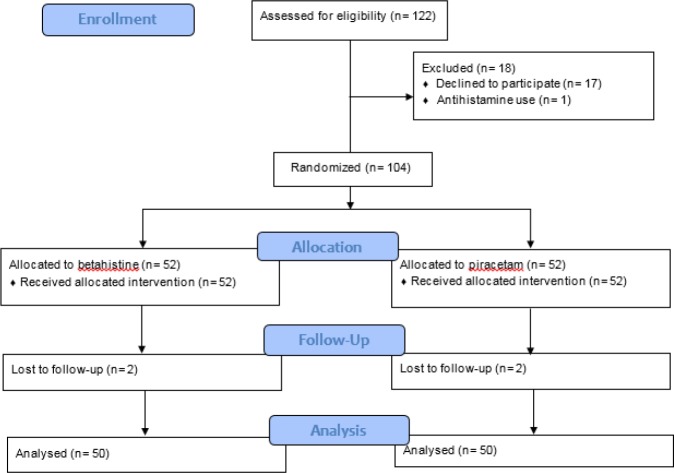
CONSORT flow diagram of the studied patients

**Table 1:** Baseline characteristics of the studied patients

**Variables**	**Groups**		**P**
	**Piracetam n=50**	**Betahistine n=50**	
**Age**	56.36 ± 12.92	53.08 ± 15.13	0.245
**Sex **			
Male	21 (42.0)	17 (34.0)	0.268
Female	29 (58.0)	33 (66.0)	
**Intensity of symptom (on presentation)**			
Vertigo	8.36 ± 1.98	8.44 ±‌ 1.63	0.844
Nausea	8.24 ± 2.50	7.52 ± 2.87	0.136
Fatigue	4.98 ±‌ 3.18	5.16 ± 2.92	0.723
**Intensity of symptom (on discharge)**			
Vertigo	1.82 ± 1.08	1.58 ± 0.73	0.397
Nausea	1.22 ± 0.55	1.26 ±‌ 0.44	0.455
Fatigue	1.42 ± 1.07	1.22 ± 0.55	0.415

Data are presented as mean ± standard deviation or frequency (%).

**Table 2:** Comparing the outcomes between the two groups 7 days after discharge from emergency department

**Outcome**	**Groups**		**P**
	**Piracetam n=50**	**Betahistine n=50**	
**Vertigo **			
Intensity	1.78 ± 1.38	1.84 ± 1.20	0.648
Success	48 (96.0)	48 (96.0)	0.691
Failure*	2 (4.0)	2 (4.0)	
**Nausea **			
Intensity	1.12 ± 0.52	1.10 ± 0.21	0.987
Success	49 (98.0)	50 (100.0)	0.500
Failure*	1 (2.0)	0 (0)	
**Fatigue **			
Intensity	1.28 ± 0.67	1.36 ± 1.41	0.437
Success	49 (98.0)	48 (96.0)	0.500
Failure*	1 (2.0)	2 (4.0)	
**secondary outcomes**			
Drug compliance	4.52 ± 0.58	4.58 ± 0.50	0.713
Adverse events	12 (24.0)	6 (12.0)	0.125

* : score ≥ 3 seven days after discharge. Data are presented as mean ± standard deviation or frequency (%).

Despite being a common presentation in both ED and general settings, the ideal treatment for vertigo is still under debate (1, 4). 

Betahistine dihydrochloride is a drug with structural similarity to histamine (13, 14). Betahistine is a strong H_3_ receptor antagonist and a weak H_1_ receptor agonist, which is relatively inactive at H_2_ receptor (13-15). Betahistine increases cochlear blood flow via H_3_ receptor antagonism (16). It also increases histamine turnover in central and vestibular nervous systems and decreases vestibular sensory input through the same mechanism. Betahistine has excitatory effects on cortical and subcortical neuronal activity by H_1_ receptor agonist (13-15).

Betahistine has been used for treating vertigo for years and its effectiveness was shown in many studies (7, 13-15, 17-22). In a Cochrane review conducted by Murdin, 16 studies comparing betahistine and placebo in different vertigo types were included. Pooled data showed that betahistine was better than placebo in reducing vertigo symptoms and the proportion of patients reporting an overall reduction of their vertigo symptoms was higher in the betahistine group (risk ratio (RR)= 1.30, 95% confidence interval (CI)= 1.05 to 1.60). However, statistical heterogeneity of data was high. The most common adverse effects were gastrointestinal symptoms and headache, which were statistically similar in both groups (16% in betahistine group and 15% in placebo group and RR= 1.03, 95% CI= 0.76 to 1.40). Authors concluded that low quality evidence suggests that betahistine may have a positive effect on reduction of vertigo symptoms and there is a low risk of adverse events associated with betahistine (13).

It has been shown that piracetam might be effective on both chronic and acute vertigo (6-10, 23). Although the exact mechanism by which piracetam affects vertigo is not determined yet, it seems to be due to an increase in vestibular compensation by exerting effects on neurotransmission and microcirculation; which result from its effects on cell membrane fluidity (8, 10, 24, 25).

There are previous studies, which have evaluated the effectiveness of piracetam in various vertigo settings. Ozdemir and Dogan compared intravenous piracetam with intravenous dimenhydrinate in the acute setting in two separate studies. Both studies showed that intravenous piracetam and intravenous dimenhydrinate have similar effectiveness in improving acute vertigo (6, 9).

In a study by Akdogan, oral piracetam and placebo were compared in chronic vertigo lasting more than 3 months. Piracetam was more effective than placebo in treating all of the symptoms (5). In a sub-analysis of data from OSVaLD study, which was conducted by Melnikov, it was shown that piracetam and betahistine combination therapy is more effective than treatment with betahistine alone in patients with peripheral vestibular vertigo(7). Rosenhal evaluated the effectiveness of betahistine in comparison with placebo for treating chronic vertigo in a multicenter, randomized clinical trial. Piracetam was more effective than placebo in decreasing frequency of vertigo episodes, interval malaise, and imbalance (10). In a study by Ince Gunal, Patients with autosomal dominant cerebellar ataxia were given intravenous piracetam. It was shown that piracetam causes improvement in posture and gait disturbances of these patients (23). 

It seems that oral piracetam is an alternative choice for treatment of peripheral vertigo and should be considered for in- and out-patient management of peripheral vertigo. 

***Limitations***

The patients and allocating physician were not blinded to the prescribed drugs. There was no placebo group in the study. We cannot rule out the probability of spontaneous recovery of vertigo, nausea and fatigue symptoms of the patients in the seven-day period after the initial presentation to ED. Because of the small sample size and differences in the settings of previous studies, it is hard to reach a definite conclusion about the role of piracetam in vertigo treatment. It seems that there is a need for larger, multicenter studies and meta-analyses for a strong conclusion.

## Conclusion:

Based on the findings, there was not any significant difference between oral betahistine and oral piracetam regarding recurrence, relieving vertigo, nausea, and fatigue symptoms, as well as adverse events 7 days after discharge from ED.
